# Multi-sensor remote sensing captures geometry and slow-to-fast sliding transition of the 2017 Mud Creek landslide

**DOI:** 10.1038/s41598-025-11399-8

**Published:** 2025-08-14

**Authors:** Alexander L. Handwerger, Pascal Lacroix, Andrew F. Bell, Adam M. Booth, Mong-Han Huang, Simon M. Mudd, Roland Bürgmann, Eric J. Fielding

**Affiliations:** 1 Jet Propulsion Laboratory, California Institute of Technology, Pasadena, 91109 USA; 2 Joint Institute for Regional Earth System Science and Engineering, University of California, Los Angeles, Los Angeles, 90095 USA; 3 ISTERRE, University Grenoble Alpes, University Savoie Mont Blanc, CNRS, IRD, UGE, Grenoble, France; 4 School of GeoSciences, University of Edinburgh, Edinburgh, EH8 9XP UK; 5 Department of Geology, Portland State University, Portland, OR 97207 USA; 6 Department of Geology, University of Maryland, College Park, MD 20742 USA; 7 Department of Earth and Planetary Science, University of California, Berkeley, CA 94720 USA

**Keywords:** Natural hazards, Geomorphology

## Abstract

Landslides pose a significant hazard worldwide. Despite advances in landslide monitoring, predicting their size, timing, and location remains a major challenge. We revisit the 2017 Mud Creek landslide in California using radar interferometry, pixel tracking, and elevation change measurements from satellite and airborne radar, lidar, and optical data. Our analysis shows that pixel tracking of optical imagery captured the transition from slow motion to runaway acceleration starting ~ 1 month before catastrophic failure—an acceleration undetected by satellite InSAR alone. Strain rate maps revealed a new slip surface formed within the landslide body during acceleration, likely a key weakening mechanism. Failure forecast analysis indicates the acceleration followed a hyperbolic trend, suggesting failure time could have been predicted at least 6 days in advance. We also inverted for the landslide thickness during the slow-moving phase and found variations from < 1 to 36 m. While thickness inversions provide important first-order information on landslide size, more work is needed to better understand how landslide subsurface properties and deforming volumes may evolve during the transition from slow-to-fast motion. Our findings underscore the need for integrated remote sensing techniques to improve landslide monitoring and forecasting. Future advancements in operational monitoring systems and big data analysis will be critical for tracking slope instability and improving regional-scale failure predictions.

## Introduction

Landslides are major hazards that have significant implications for the natural and human-made environment worldwide^1–3^. Landslides are most often triggered by rainfall^4–6^ and earthquakes^7–9^; however other factors, such as poor land management^10–12^, permafrost degradation^13^, and rapid glacier-retreat^14,15^ add to the landslide problem. Although the landslide problem is well known, there is still much to uncover about landslide processes, and in particular those related to landslide prediction. More specifically, questions remain relevant to landslide prediction such as when and where will a landslide occur or suddenly accelerate and how will it move downslope once it fails? These questions are relevant to understanding landslides, particularly through the lens of hazard mitigation and response, and for developing effective mitigation strategies to minimize their impacts.

Predicting when and where natural hazards such as landslides will occur has been a major focus of research for decades^16–20^. This prediction seeks to provide information on the location, timing, and size of hazardous events. Two main approaches have been used for landslide prediction, either statistically through the analysis of inventories of rapid landslides to decipher their location and triggering conditions^21,22^, or deterministically by measuring slow motion that precedes runaway acceleration and catastrophic failure^16,23,24^. The latter approach has benefited from modern remote sensing techniques, such as interferometric synthetic aperture radar (InSAR) and pixel offset tracking of SAR and optical images, to identify and monitor landslide motion^25–27^. A number of studies have shown it is possible, although retrospectively, to use satellite data to detect precursory accelerations leading up to catastrophic failures, and in some cases identify the timing of runaway failure^18,25,26,28^. These same remote sensing data have also been used to develop regional-scale inventories of slow-moving landslides^29–35^, and near-operational tools to track landslide kinematics^36,37^, all of which help identify unstable hillslopes and further improve the understanding of landslide mechanisms.

In addition to the important kinematic measurements, remote sensing datasets also provide critical information on landslide geometry. In particular, the surface extent can be easily identified by examining deformation maps in combination with high resolution digital elevation models (DEMs) and/or optical imagery. Yet, it remains a major challenge to constrain the subsurface geometry of landslides before they evacuate material from the hillslope and create measurable scars, which is when it’s most important to gain this information. A landslide’s geometry results from the stresses acting on the hillslope and subsequently determines stress distribution on the failure surface^38–40^, impacts the groundwater hydrology^5,41,42^, and may be useful in predicting landslide runout^43,44^. Traditionally, the thickness and volume of landslides have been estimated through geometric scaling relations^45–48^, extrapolation of borehole observations, geophysical measurements that assume the landslide basal shear zone at depth is occurring at a lithological contact characterized by a contrast of either impedance or resistivity^49,50^, or through estimation of the landslide sliding surface geometry by inversion of 3D displacement fields obtained from remote sensing, assuming a specific rheology^51–55^. Few studies^56^ have applied these methods on slow landslides that evolved into rapid landslides, leading to a better opportunity to validate the volume estimations.

While satellite observations provide the best opportunity to detect and predict landslides broadly, there are still many challenges that must be overcome before a reliable operational system can be developed to detect and characterize hazardous landslides^57^. The variety of landslide types, sizes, and behaviors, slope orientations, weather conditions, and more can contribute to challenges for landslide monitoring and prediction. As a result, using one single monitoring technique is often insufficient for capturing the full range of landslide processes and behaviors leading up to catastrophic failure. For instance, InSAR techniques are challenged in steep topography, limited by only providing 1D measurements, and cannot reliably measure large ground deformations (> dm-scale per image pair) where phase unwrapping errors result in aliased or erroneous data^58–62^. Pixel tracking of optical images overcomes two of these limitations. It can measure large ground displacements, and the typical vertical look angle of these instruments means landslides cannot fall into an instrument shadow in steep terrain, as is the case for InSAR. Additionally, it resolves the two components of horizontal motion, rather than one component in the line-of-sight. However, pixel tracking techniques feature lower accuracy than InSAR (limited to ~ 1/10 pixel size) and are limited by cloud-cover that can drastically reduce the number of available images^36,63,64^. Pixel tracking of SAR data can also be useful in cases of larger deformations^65–67^. On the other hand, repeat lidar or other 3D representations of the earth’s surface allow for 3D surface displacements to be measured, but with lower precision than InSAR and lower temporal resolution^53,68–70^. Thus, the combination of pixel tracking, InSAR, and repeat digital elevation model techniques provide a way to capture a broader range of processes from slow (cm/yr) to fast (cm/day) landslides in three dimensions.

Here we present new results that use exclusively remote-sensing data to examine the slow-to-fast sliding transition of the 2017 Mud Creek landslide, Big Sur Coast, California, USA. By combining observations from InSAR, optical, and lidar we (1) precisely identify the location and extent of the landslide, (2) estimate the subsurface geometry before catastrophic collapse, and (3) accurately predict the time to runaway failure before destabilization occurred. We hypothesize that the transition from slow-to-fast sliding is controlled by a progressive material weakening and shear crack growth. We also show that InSAR is well suited for measuring years of slow (cm/yr) seasonal motion but fails to capture the transition to runaway failure, whereas pixel tracking captures the period of fast motion (m/yr) as the landslide rapidly accelerated toward failure but struggles to detect the slower period of motion. We conclude by discussing the viability of remote sensing for real-time landslide prediction.

## The 2017 Mud Creek landslide, Big Sur, California

The Mud Creek landslide located on the Big Sur Coast, California, failed catastrophically on May 20, 2017 and destroyed part of the California State Highway 1 (CA 1). The failure occurred on a dry day near the end of the extreme 2016–2017 wet season, but was the culmination of years of slow-motion^59,71,72^. Fortunately, no lives were lost due to this event, but CA 1 remained closed for more than a year due to challenging reconstruction efforts. The road repair cost more than $50 million and the road closure led to major costs to the local economy^73,74^. The Big Sur Coast has many other active slow-moving landslides that continue to slide slowly^31,35^.

Several previous studies have examined the Mud Creek landslide using satellite and airborne InSAR, lidar, and structure from motion (SfM) data^59,71,72^, as well as mechanical models^75^ and community detection methods^76^. These studies concluded that Mud Creek failed catastrophically as a result of unusually high rainfall in preceding months (i.e., high pore-water pressure) and exhibited slow motion for at least 8 years prior to failure. The surface area of the landslide (area ~ 200,000 m^2^) was larger than the section that ultimately collapsed (area ~ 80,000 m^2^)^59,72^, suggesting that slip localized during or before failure. The vertical change of the catastrophic failure ranged from a few meters up to -57 m, and the mobilized volume was between 2.5 × 10^6^ and 3.0 × 10^6^ m^3^^72^.

Given the years of slow motion that preceded the collapse of Mud Creek, this landslide presents an opportunity to explore the use of remote sensing data to forecast catastrophic failure and to test methods for estimating the subsurface geometry. While previous work using satellite InSAR^59,71^ had concluded it was not possible to accurately forecast the timing of failure due to challenges related to InSAR techniques (i.e., low coherence, unwrapping errors and phase aliasing from large displacement), other methods such as pixel offset tracking can better perform in cases of large displacement^18,77^. Here, we combine the use of InSAR and pixel offset tracking of lidar and optical data to investigate the pre-collapse behavior and geometry of the Mud Creek landslide (Methods). We now find a clear, hyperbolic acceleration of the landslide in the weeks before failure, which would have allowed for issuing a prediction of impending failure.

## Results

In this paper, we construct new remote sensing datasets and reanalyze published datasets^59^ for the Mud Creek landslide (Methods). Our analysis focused on the 8-year period between 2009 and 2017. We analyze datasets of landslide motion and geometry derived from the Copernicus Sentinel-1A/B SAR satellites and the NASA Jet Propulsion Laboratory Uninhabited Aerial Vehicle Synthetic Aperture Radar (UAVSAR) airborne system, airborne lidar from the U.S. Geological Survey’s 3D Elevation Program (USGS 3DEP), and satellite optical data from PlanetScope. We focus our analysis on the landslide geometry and kinematics to better understand how the landslide evolved through time and to determine if we could have predicted its catastrophic collapse.

### Landslide extent and behavior

**Fig. 1:**
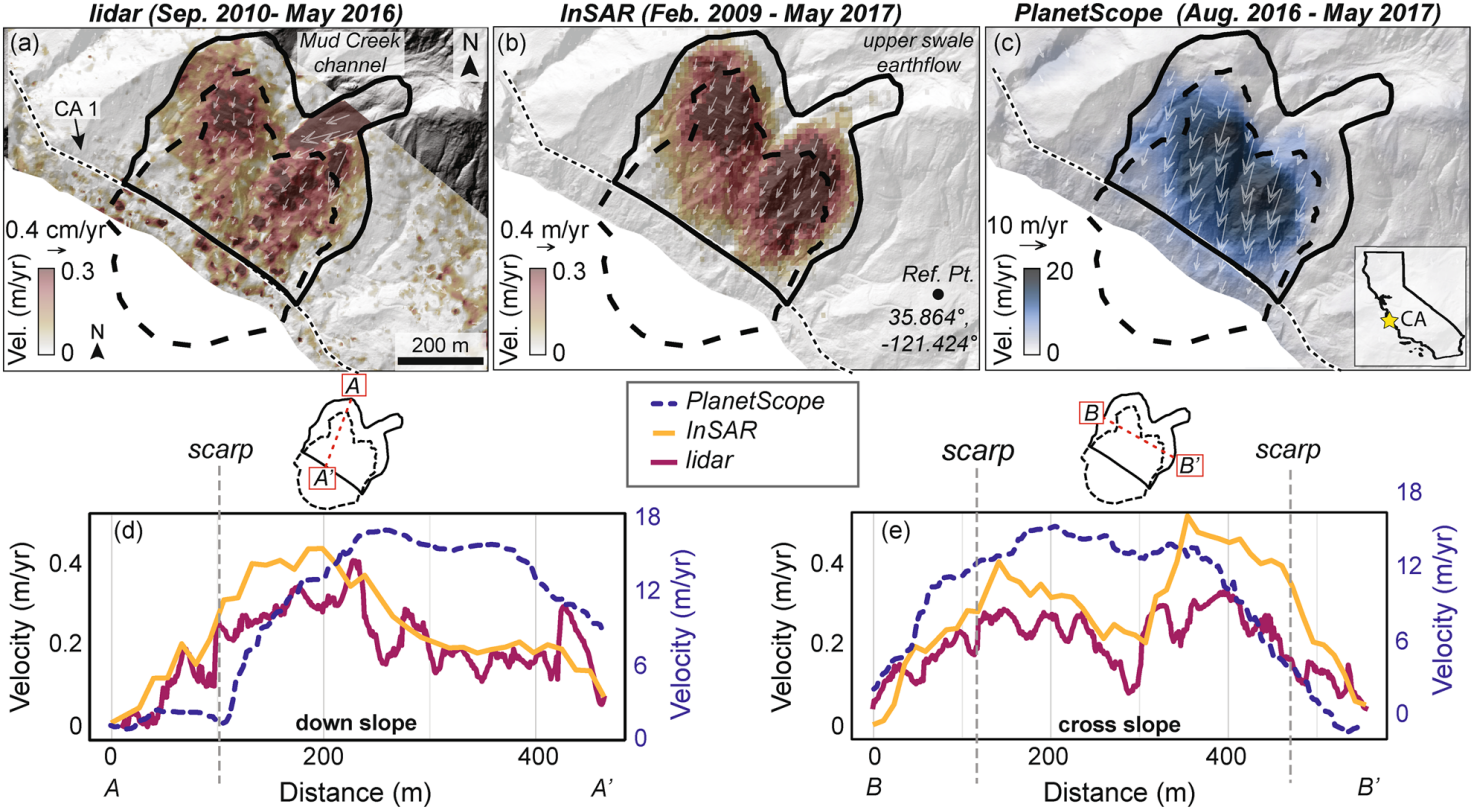
Horizontal surface velocity of Mud Creek landslide. Velocity measured from (**a**) lidar pixel tracking (Sep. 2010—May 2016), (**b**) InSAR (Feb. 2009–May 2017), (**c**) PlanetScope pixel tracking (Aug. 2016–May 2017). Velocity maps and velocity vectors are draped over the pre-catastrophic failure hillshade of topography. Solid black line shows the pre-catastrophic failure landslide boundary. Dashed black line shows the catastrophic failure area including the landslide scar and deposit. Reference pixel (Ref. Pt.) corresponds to a not-moving area outside the landslide. Thin dashed black line shows California State Highway 1 (CA 1). The Mud Creek channel is labeled in (**a**). Velocity profiles for the (**d**) downslope and (**e**) cross slope directions. Insets above (**d**,**e**) show the location of the profiles. Note the large difference in velocity magnitude between lidar/InSAR and PlanetScope, due to the latter capturing the rapid acceleration prior to failure (see Fig. 2). InSAR data were previously published in Ref.^59^).

The Mud Creek landslide consisted of two active zones that spanned across the Mud Creek channel, and an earthflow (referred to as “upper swale earthflow” by Warrick et al.,^72^) in the northeastern part of the slope. Figure 1 shows the horizontal velocity of Mud Creek over different time periods measured with three independent datasets (additional velocity maps shown in Supplementary Figure S1). The thick black line shows the boundaries of the slow-moving landslide, including the upper swale earthflow. The dashed black line shows the outline of Mud Creek that collapsed catastrophically on May 20, 2017, including the landslide deposit which expanded into the coastal zone. The velocities measured from lidar and InSAR over roughly the same multi-year time period show overall good agreement within the main landslide boundaries. Between 2009 and 2017, Mud Creek was moving < 1.2 m/yr, with rates that varied depending on the landslide zone (Supplementary Table S1). The median horizontal velocity within the boundaries of the active landslide measured with lidar was 0.18 ± 0.15 m/yr (± 1 standard deviation). The median horizontal velocity measured with InSAR was 0.19 ± 0.11 m/yr. The fastest moving part of the main landslide body occurred near what ultimately became the headscarp of the catastrophic failure Interestingly, the lidar data showed that the upper earthflow was also moving relatively fast (0.4 m/yr), but the InSAR data did not capture significant motion in this zone, possibly due to phase unwrapping errors or differences in the timing of motion. We note that the InSAR data analyzed here were previously published in Ref.^59^. We did not truncate the data to match the temporal span of the lidar because we wanted to use all available pre-catastrophic failure data.

**Fig. 2:**
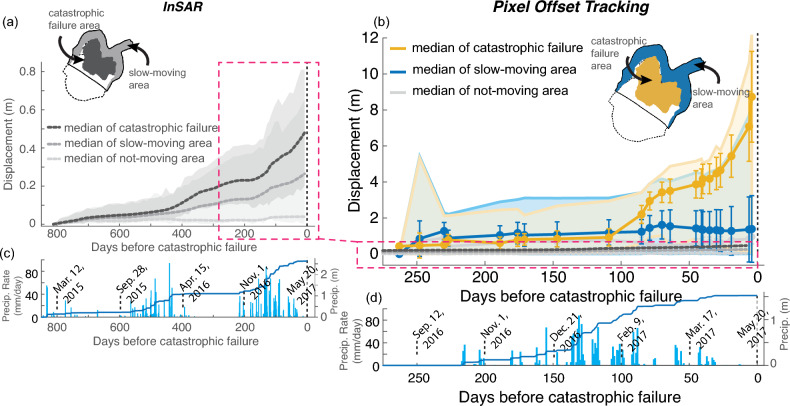
Displacement and precipitation time series. (**a**) Downslope displacement time series (see Methods) from InSAR (Sentinel-1 descending track 42) 800 days prior to catastrophic failure. (**b**) Horizontal displacement time series from PlanetScope pixel tracking ~ 275 days prior to catastrophic failure. InSAR data from (**a**) are also plotted on (**b**) within the magenta box. (**a**,**b**) Thick lines show the median value for the catastrophic failure area, the slow-moving area, and a not-moving area off the landslide. Error bars in (**b**) show standard deviation. Shaded region shows the full range of values measured for each landslide area (see inset maps). (**c**,**d**) Precipitation rate and cumulative precipitation time series. Note the large difference in magnitude between the displacement measured from InSAR and PlanetScope in the catastrophic failure area, likely caused by severe phase aliasing problems in the last 100 days before failure (see text for discussion). InSAR data were previously published in Ref.^59^.

To better compare the spatially varying kinematics, we delineated the landslide into 3 areas: (1) the *catastrophic failure area*, which was the part of Mud Creek that failed catastrophically, (2) the *slow-moving area*, which was the part of Mud Creek that moved slowly but did not collapse, and (3) the *not-moving area*, which is stable ground located outside of the landslide body (see Fig. 2 for the location of the named areas). The *not-moving* area was used for comparison and to better understand the uncertainties in the velocity measurements. Between 2009 and 2017, the median horizontal velocity of the catastrophic failure area*,* slow-moving area, and not-moving area measured with lidar was 0.23 ± 0.07 m/yr (± 1 standard deviation), 0.15 ± 0.22 m/yr, and 0.05 ± 0.030 m/yr, respectively. The median horizontal velocity of the three areas measured with InSAR was 0.30 ± 0.081 m/yr, 0.12 ± 0.097 m/yr, and 0.019 ± 0.0071 m/yr, respectively.

While the InSAR- and lidar-based measurements agree over the multi-year period of slow motion, the PlanetScope data reveal a drastic change in the landslide deformation that occurred between August 2016 and May 2017. The landslide velocity increased during this time period to rates up to 17 m/yr (with spatial variation). The median horizontal velocity during was 7.4 ± 5.4 m/yr (± 1 standard deviation). The highest velocities occurred within the part of the slope that ultimately failed catastrophically. The median horizontal velocity of the catastrophic failure area*,* slow-moving area, and not-moving area was 14.5 ± 3.94 m/yr, 1.99 ± 2.62 m/yr, and 1.48 ± 0.57 m/yr, respectively. We note, there was little-to-no measured motion prior to February 2017 (Fig. 2), however the spatial resolution of PlanetScope (~ 3 m) limits the theoretical accuracy of pixel tracking to ~ 30 cm per image pair, and closer to 60 cm in reality^18^. The uncertainty in our pixel tracking measurement had a mean of 58 cm (Methods).

We also examined the time-dependent motion of the Mud Creek landslide using Sentinel-1 satellite InSAR and pixel tracking of PlanetScope data. Note that while we were able to retrieve full 3D motion for the average velocity maps derived from InSAR, this retrieval is not possible for time series analysis due to differences in the timing of acquisition of ascending, descending, and UAVSAR data. For the InSAR time series analysis we present the 1D LOS displacement projected onto the downslope direction (see details in Ref.^59^. Satellite InSAR data going back 800 days before the catastrophic failure showed that Mud Creek exhibited seasonal motion driven by rainfall (Fig. 2a). The catastrophic failure area moved faster and further than the slow-moving area. The median downslope displacement of the catastrophic failure area was 0.49 ± 0.12 m (± 1 standard deviation), the median displacement of the slow-moving area was 0.26 ± 0.15 m, and the median displacement of the not-moving area was 0.04 ± 0.03 m (Supplementary Table S1). The landslide exhibited increasing seasonal displacement over the study period as the region transitioned from historic drought conditions in 2015 to record rainfall in 2017. A more detailed analysis of the seasonal behavior of the landslide is described in Ref.^59^.

The pixel tracking time series from PlanetScope data show that Mud Creek started to accelerate ~ 85 days (between January 31 and February 24, 2017) before catastrophic failure and exhibited little to no detectable motion (within uncertainty) prior to then (Fig. 2b). The median horizontal cumulative displacement of the catastrophic failure area was 8.7 ± 2.5 m (± 1 standard deviation), the median horizontal cumulative displacement of the slow-moving area was 1.4 ± 1.9 m, and the median horizontal cumulative displacement of the not-moving area was 0.97 ± 0.40 m. The catastrophic failure area appears to have moved ~ 3 m in between February 9, 2017 and March 17, 2017 before temporarily slowing back down. Then, starting ~ 30 days before failure, the catastrophic failure area started to exhibit the runaway acceleration behavior that is characteristic of progressive slope failure^78^. This type of failure pattern suggests we could have predicted the timing of landslide collapse (see Results: *Forecasting Time-of-Failure using PlanetScope Pixel Tracking*). While there is a large range of motion exhibited by Mud Creek, the collective behavior of each zone differs in important and significant ways. The catastrophic failure area shows a runaway acceleration pattern while the slow-moving area does not. Both areas show median displacement greater than the not-moving area, however there is overlap in the range of values for the slow-moving area and not-moving area in the final weeks before catastrophic failure.

Our findings also show a major discrepancy between the motion measured by InSAR and pixel offset tracking over the final acceleration period (Fig. 2). The InSAR data have significantly underestimated the large displacements during the last ~ 100 days before failure, most likely due to phase unwrapping error known as phase aliasing^62^, and thus provide unreliable results. We will discuss the implications of this result in the Discussion section.

**Fig. 3:**
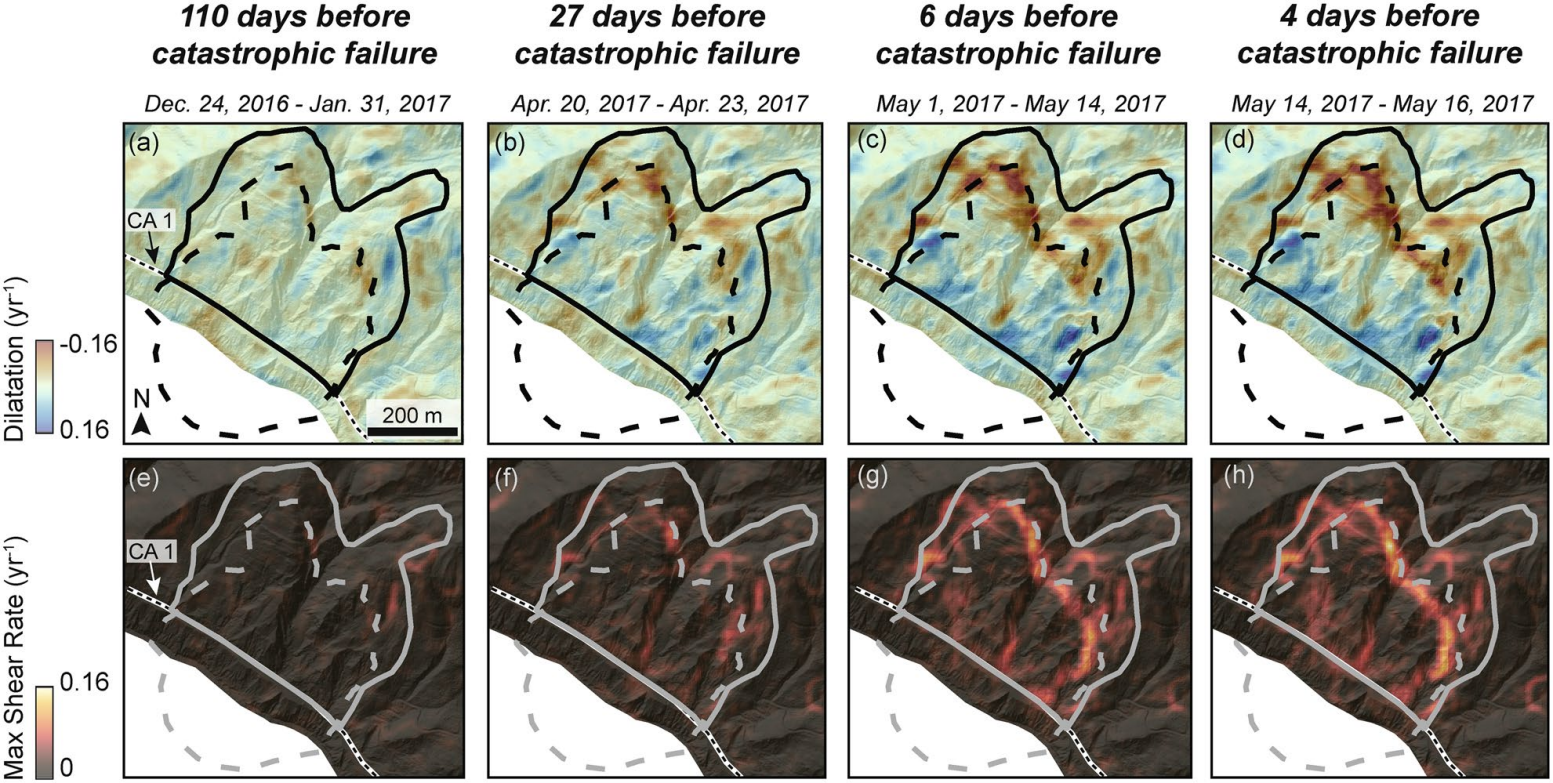
Strain rate maps from PlanetScope pixel tracking displacement time series. Strain rate maps are draped on pre-catastrophic failure lidar hillshade. (**a**–**d**) Dilatation and (**e**–**h**) maximum shear rate from sequential image pairs 110 days, 27 days, 6 days, and 4 days before catastrophic failure. Solid black or gray line shows the pre-catastrophic failure landslide boundary. Dashed black or gray line shows the catastrophic failure area including the landslide scar and deposit. Black and white dashed line shows the location of California State Highway 1 (CA 1). Note that these strain rate maps were selected to show the development of the landslide slip-surface boundaries prior to catastrophic failure of the landslide.

We also examined the spatial evolution of the Mud Creek slope using strain rate maps derived from the PlanetScope data (Fig. 3; see Methods). The strain rate maps show strain localization starting a month prior to failure. The dilatation shows extension (negative values) in the upper headscarp, where the slope was pulling apart, and contraction (positive values) at the slide toe, where the slope was compressing along CA 1. The max shear rate represents the maximum shear strain (i.e., when a fracture is 45 deg away from the principal strains), and it clearly shows localization starting 27 days before catastrophic failure with strain clearly concentrating along the boundaries of what eventually became the catastrophic failure scar by 6 days before catastrophic failure. Note that strain rate time series are not possible with InSAR due to the 1D LOS measurement in the time series result.

### Forecasting time-of-failure using PlanetScope pixel tracking

**Fig. 4:**
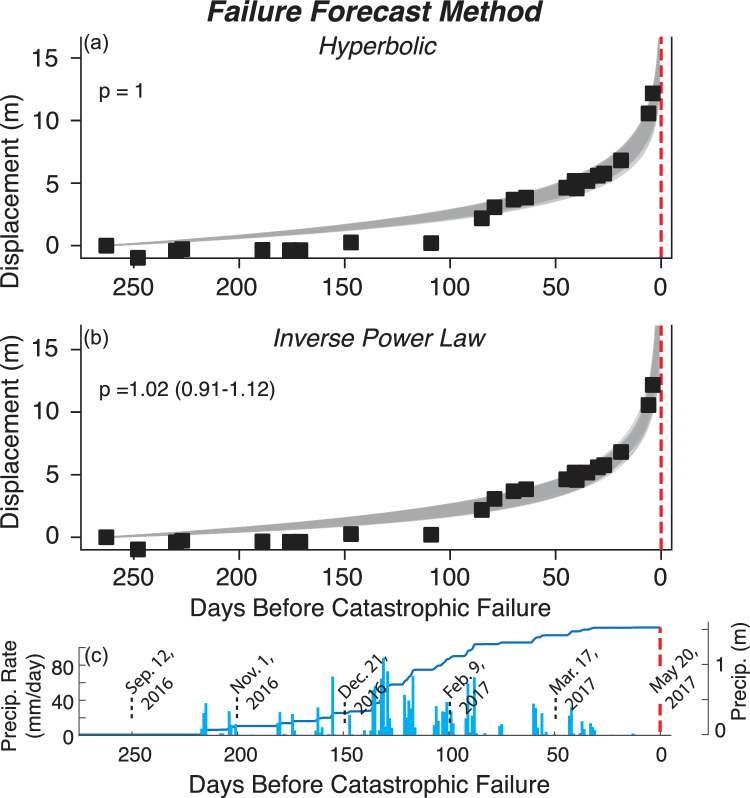
Failure Forecast method applied to landslide displacement time series measured from PlansetScope. (**a**) Hyperbolic and (**b**) inverse power law model applied retrospectively to landslide displacement at a representative area (location: 35.865425°, -121.429479°). The gray lines represent 500 samples from the posterior parameter distributions after Markov Chain Monte Carlo method (MCMC) sampling. (**c**) Precipitation rate and cumulative precipitation time series. Note that *p* is the exponent in Eq. (3).

The displacement time series measured from PlansetScope data preceding the catastrophic failure was modeled using a Bayesian non-linear Gaussian regression method, with cumulative displacement evolving according to the integral of Eq. (3). Firstly, this was undertaken as a retrospective analysis, where the failure time is known, and fixed. Results of this analysis are shown in Fig. 4. In each case, the lines represent 500 samples from the posterior parameter distributions after Markov Chain Monte Carlo method (MCMC) sampling. Figure 4a shows the hyperbolic model (*p* = 1) applied to the displacement time series at a representative area (location: 35.865425°, − 121.429479°) within the Mud Creek landslide. Figure 4b shows the same data with the inverse power-law models (*p* in Eq. (3) can take a range of values). Both power-law and hyperbolic models provide a good fit to the data. For the power-law model, the median *p*-value is 1.02 with the 89% range from 0.91 to 1.12.

**Fig. 5:**
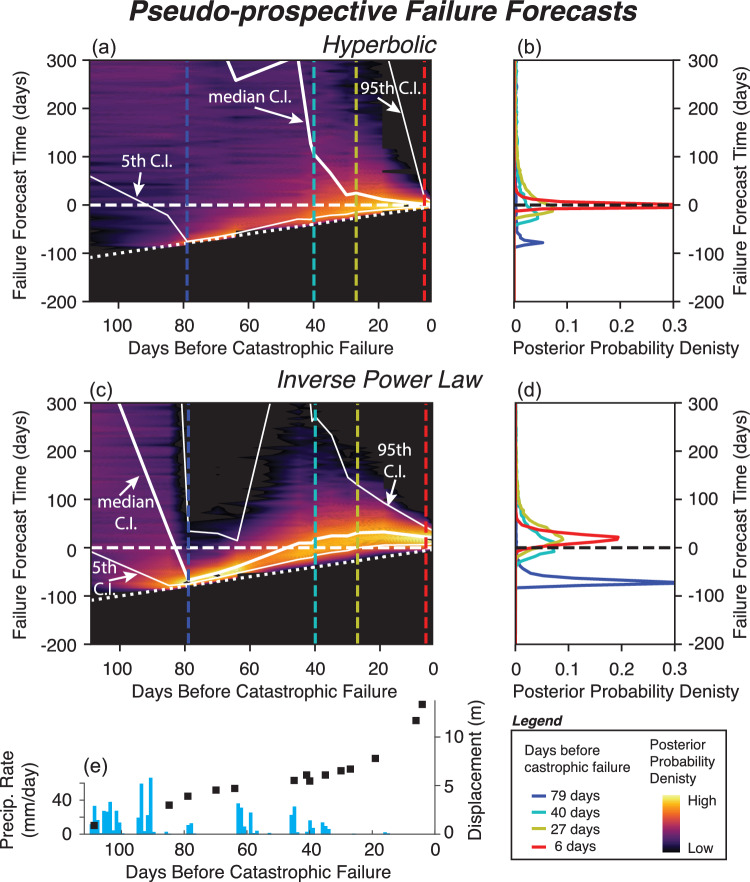
Evolution of the posterior probability density of the landslide failure time. The color scale shows the posterior probability density for the (**a**) hyperbolic and (**c**) inverse power law models. The solid white lines indicate the median and 5% and 95% credibility intervals (labeled as C.I.) of the posterior. The C.I. lines show when and how the forecast starts to converge. The horizontal white dashed line indicates the true value of landslide failure time (failure forecast time = 0), and the dotted white line indicates actual time at which hindcast is made. (**b**,**d**) Posterior probability density functions at times indicated by correspondingly colored dashed lines (**a**,**c**). (**e**) Precipitation and landslide displacement time series for comparison.

We also performed repeated pseudo-prospective forecasts, where the failure time is unknown, and data is added incrementally as if it were becoming available in real-time. This analysis reveals the evolution of parameter posterior distributions as the sequence progresses, and provides insights into the potential for using displacement data for forecasting the timing of similar landslide events in the future. Figure 5 shows examples of ‘pseudo-prospective’ modeling of the landslide displacement time series at a representative area measured from PlanetScope data at different stages through the sequence (1) 79 days before failure, (2) 40 days before failure, (3) 27 days, and (4) 6 days before failure. Figure 5 illustrates the temporal evolution of the posterior probability distributions for the forecast failure time for the second half of the sequence. As the failure model only accounts for a single monotonic increase in displacement rate, the increase in these rates between 80 and 64 days before failure results in a ‘false alarm’. For these forecasts, the models which best fit the data are those with low values of the failure time, and relatively low (but non-zero) probabilities are assigned to the actual failure time. The false alarm is stronger in the case of the inverse power-law, where the displacement rate increase can be accommodated by covariance between the values of *p* and the failure time. As rates slow again, such short failure times are no longer likely. Confidence in the forecast increases as more data become available through the sequence and the distribution of possible failure times converge toward the true failure time. For the hyperbolic model, the forecast converges toward the real failure time with high probability between 27 and 6 days before failure. For the inverse power-law model, the forecast converges toward the real failure time but ultimately predicts the highest probability of failure a few days after the real failure time.

### Subsurface geometry

**Fig. 6:**
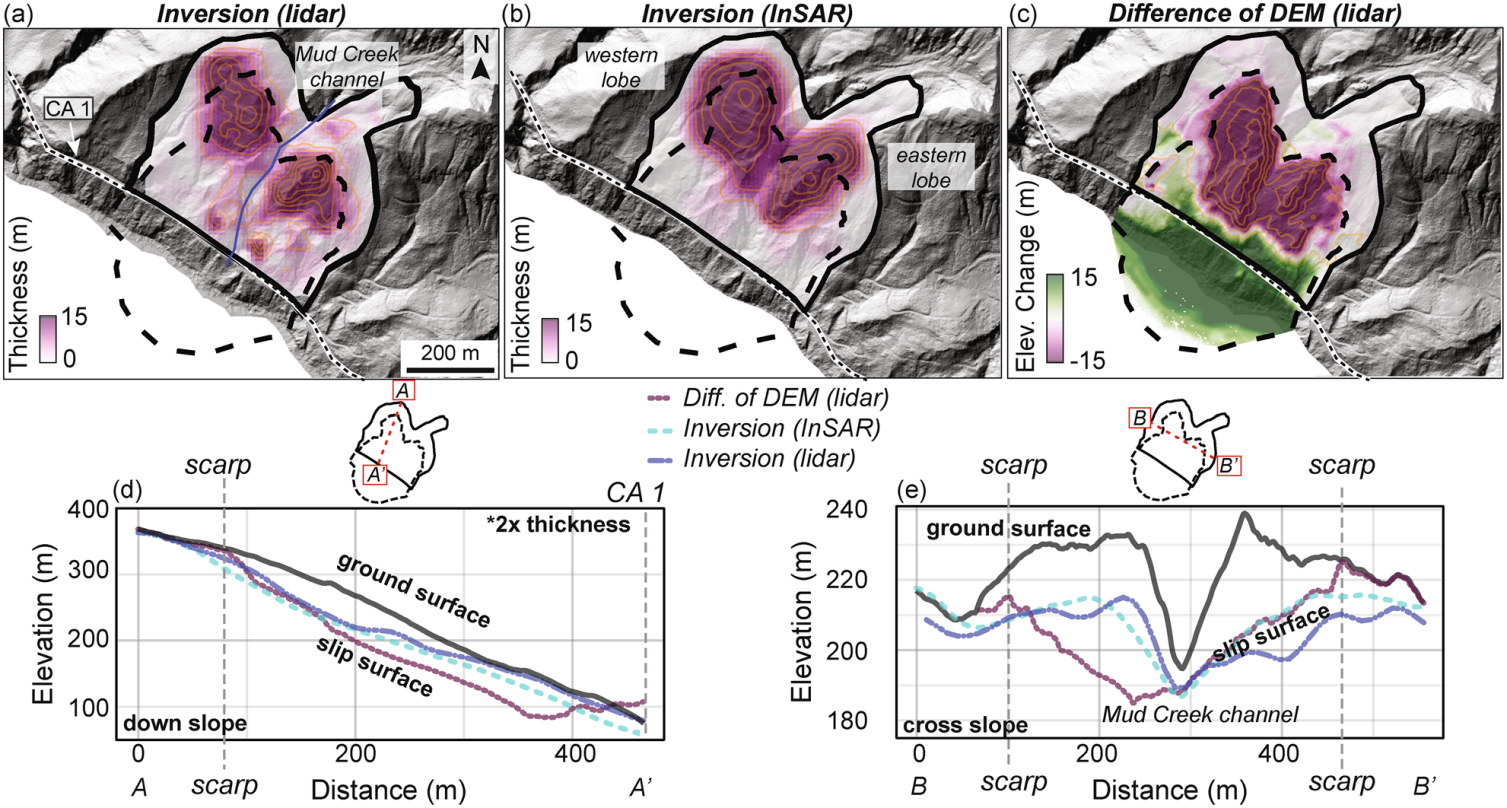
Landslide thickness and lidar change maps. Landslide thickness inversion maps from (**a**) lidar surface velocity and (**b**) InSAR surface velocity maps. Orange lines show 5 m thickness contours in (**a**,**b**). (**c**) Vertical elevation change derived from pre- (2016) and post-collapse (2018) lidar point cloud. Orange lines show 10 m contours in (**c**). Positive values correspond to material deposit after failure. Maps are draped on pre-collapse lidar hillshade. (**d**–**e**) Down slope and cross slope ground surface and slip surface elevation profiles. The down slope profile for the slip surface is scaled by 2 × thickness in the vertical direction. Dashed vertical gray lines show where the profile crosses the catastrophic failure scarp. Insets show location of profiles.

We also estimated the subsurface geometry of the Mud Creek landslide prior to its catastrophic failure using volume conservation from both lidar and InSAR measurements (see Methods section). The thickness inversion estimates from lidar (Fig. 6a) and InSAR (Fig. 6b) show spatial variability with the thickest parts downslope of the catastrophic headscarp. The inferred thickness maps reveal two deep lobes, on either side of the Mud Creek channel. The shape of the lobes are similar to the catastrophic headscarp shape. The inversion also predicts significant thickness outside of the catastrophic failure area and within the slow-moving area. Thus a significant amount of active material remains in the slow-moving area, which continued to move at rates > 1.4 m/yr through the first half of 2024^79^ (https://www.usgs.gov/media/images/images-show-same-views-mud-creek-landslide-california-and-after-winter-2024-season).

For the lidar inversion, the landslide had a mean thickness of 12 m and maximum thickness of 36 m within the catastrophic failure area (Supplementary Table S2). Including the full moving landslide area (i.e., catastrophic failure area + slow-moving area), the mean thickness is 7 m and maximum thickness remains at 36 m. For the InSAR inversion, the landslide had a mean thickness of 15 m and maximum thickness of 32 m within the catastrophic failure area, and a mean thickness of 8 m and maximum active thickness of 32 m when including the slow-moving area. We also plotted basal topography profiles to examine the shape of the inferred basal sliding surfaces. The downslope profile (Fig. 6d) shows a concave up slip surface with the thickest part close to the catastrophic headscarp. The cross slope profile (Fig. 6e) shows a more complex topography with both convex up and concave up sections in the western lobe, while the eastern lobe shows a more typical concave up shape.

We also performed lidar differencing (referred to as difference of DEM, or DoD) using pre- and post-catastrophic failure lidar data collected in September 2016 and March 2018, respectively (see Methods section). A negative change in elevation corresponds to material lost during failure and the vertical distance may approximate the landslide thickness. Focusing only on material that was depleted during failure, we find the lidar differencing reveals a mean elevation change of − 22 m (decrease in elevation after catastrophic failure) and maximum elevation change of − 53 m within the catastrophic failure area. The difference of DEM method also shows the landslide deposit had elevation gain and the expanded coastline that resulted. We note that the March 2018 DEM was acquired nearly 1 year after the catastrophic failure of Mud Creek and during this time there was significant human-impact to the slope area in order to rebuild the road, and more material could have slid downslope leading to increased elevation change values. Nonetheless, by comparing the inferred thickness with the measured elevation change, we find that overall, the inferred values underestimate the measured elevation change within the catastrophic failure scarp. The area with major elevation changes also extends more than ~ 100 m downslope from the inferred slip surface of the western lobe. There is better overall agreement between the inverted thickness values and measured elevation change in the eastern lobe of Mud Creek. Lastly, we estimated landslide thickness using the geometric scaling relations proposed by Ref.^55^ and found thickness estimates between 6.5 and 9 m (Supplementary Table S2). This geometric approach underestimates the Mud Creek thickness on average and also does not capture spatial variations in thickness. A particular challenge of geometric scaling estimates for Mud Creek was that the landslide changed in surface area during the catastrophic failure.

We calculated the landslide volume using the inversion results and the lidar differencing for the full moving landslide area and the catastrophic failure area. For the lidar inversion, the landslide had an inferred volume of ~ 1.5 × 10^6^ m^3^ and ~ 9.7 × 10^5^ m^3^, respectively. For the InSAR inversion, the landslide had an inferred volume of ~ 1.9 × 10^6^ m^3^ and ~ 1.2 × 10^6^ m^3^, respectively. For the lidar difference of DEM, the landslide had a measured minimum volume of ~ 2.1 × 10^6^ m^3^ in the catastrophic failure area scar. The lidar and InSAR inversion results underestimated catastrophic failure area volume by a factor of ~ 2.2 and ~ 1.75, respectively.

## Discussion

Our results demonstrate that the Mud Creek landslide transitioned from slow (cm/yr) to fast (cm/day) motion in the final weeks before catastrophic failure. While such accelerations are expected en route to catastrophic landslide failure^18,80^, the timescale over which this transition happened at Mud Creek had not been quantified. Previous work^59,71^ that used InSAR data alone, missed meters of displacement between February and May 2017, and as a result, did not capture the runaway failure pattern revealed by the PlanetScope data. The most likely reason for this measurement error is related to phase aliasing from unwrapping errors. While phase aliasing and unwrapping errors are well-known issues in InSAR, it can be challenging to constrain these errors without additional measurements of ground surface motion. Recent work by Pepin & Zebker^62^ explored the impacts of phase aliasing using both synthetic and real examples and concluded that (1) aliasing artifacts are not easily recognized and (2) there is no one best approach for minimizing these artifacts. Strategies to reduce phase aliasing rely on multiple types of data (e.g., SAR and optical from multiple satellites) and techniques and instruments (e.g., InSAR, pixel tracking, GNSS, extensometers) to measure ground surface motion. Based on our findings, when working in areas without ground-instrumentation we recommend using both InSAR and pixel tracking methods simultaneously to capture both slower and faster motion more accurately. Future work should be aimed at developing methods to merge the multi-sensor datasets into a single time series.

With the improved landslide displacement measurements from PlanetScope pixel tracking time series, we investigated whether these data could have been used to forecast the failure time. Our results indicate that the landslide accelerated toward catastrophic failure in a predictable way. The acceleration of the landslide over time follows a power-law relationship, with an exponent *p* ~ 1.0 – consistent with the hyperbolic failure model^16,24^. The hyperbolic failure model is thought to be related to crack growth whereas crack nucleation follows an exponential trend, which suggests crack growth processes may be relevant in the case of Mud Creek^24,81,82^. However, empirical evidence on how closely natural failures follow these theoretical models remains limited. Enhancing landslide forecasts will require a systematic analysis of new remotely sensed and various types of ground-based data, to better constrain prior assumptions about model structure and parameter values under different conditions, e.g., deformation versus seismicity rate, deep versus shallow failures^16^.

Forecasting slope failure timing and evolution is inherently probabilistic. The Bayesian Markov Chain Monte Carlo (MCMC) approach used in this study provides valuable insights into the failure process and offers several advantages^16,83^. It efficiently integrates prior knowledge about model structure and parameter values, leading to more accurate early-stage forecasts and enabling the estimation of credibility intervals for posterior parameter distributions. Additionally, this method highlights significant deviations from the expected behavior of preferred models. As the natural world will only ever approximate simple models such as that described by Eq. (3), false alarms and early failures are to be expected. Our approach allows for the development of more complex time series models (outside the scope of this work), incorporating factors such as seasonal or rainfall-dependent variations, or transitions from exponential to power-law acceleration. These ‘next-generation’ models may help to reduce false alarm rates. Improved empirical or theoretical constraint on reasonable durations of the failure process or minimum displacement at the initiation of failure, would also improve forecast quality. In addition to time dependent changes there are spatial changes that may need to be accounted for. Our remote sensing observations reveal that failure progression is not uniform across the entire landslide surface. To better understand how different sections of the slope evolve over time, we can use various displacement measures—such as mean, median, or maximum displacement of the deforming area —or an ensemble approach based on randomly selected moving pixels. In addition, strain rate data (Fig. 3) allow for detection of spatial changes in the landslide extent during the accelerating phase which may provide key information on certain areas for further investigation. We recommend future work focus modeling all parts of the slope as well as random selections of pixels but a full analysis of the implications of using different displacement metrics. Further testing and case studies are needed to refine these techniques, but our findings here, along with other recently published cases^18,26,56,80,84,85^ show promise in future development of real-time warning systems.

The 3D surface velocity measurements from lidar and InSAR allowed us to estimate the pre-catastrophic failure subsurface geometry of the Mud Creek landslide. Knowledge of the subsurface geometry prior to catastrophic failure is important because it allows for calculations of landslide stresses and improves estimates of landslide runout and erosion rate^44,47,86^. Yet, measurements of subsurface geometry are possibly the most difficult to make and most of what we know from other landslides comes from individual boreholes that may fail to capture spatial variation in subsurface properties. Our results showed that we were able to achieve estimates that were of similar magnitude, within a factor of ~ 2, to those measured by differencing the pre- and post-catastrophic failure lidar. While we are encouraged by the general agreement between our estimates and measurements, our results also show there are notable discrepancies in all three datasets that must be considered (Supplementary Fig. S2). For instance, there are clear differences in the shape and magnitude of the landslide thickness shown in Fig. 6, which could point to real differences in slip surface location or extent as the catastrophic failure evolved or to errors or biases in the data and methods themselves. These apparent differences may result from a variety of reasons, such as error in the 3D surface velocity results, DEM errors, or invalid assumptions (e.g., rigid block sliding, one slip surface, no erosion or deposition) used in the volume conservation approach (see Methods). In addition, our estimates from volume conservation account for the entire landslide body, only part of which ultimately fail catastrophically. We observed that the mapview geometry of Mud Creek changed during the transition to catastrophic failure and so it is plausible that the subsurface geometry may have also changed. Specifically, the catastrophic slip surface appears to have propagated downslope from the actively slow-moving area, both in terms of the landslide’s areal extent and slip surface geometry (Fig. 6). These types of real changes and measurement uncertainties are confounding. Yet we are encouraged that as 3D surface velocity measurements become increasingly available there will be more cases around the world where these methods can be calibrated and validated. Such improved estimates of landslide slip surface shape should help to constrain spatial variations in thickness that can be used to infer stresses and develop mechanistic models to improve forecasting of landslide motion, which is not possible when only volume is estimated from traditional geometric scaling relations.

The catastrophic failure of the Mud Creek landslide highlights the potential of slow-moving landslides to rapidly accelerate, necessitating a deeper understanding of the mechanisms that control this behavioral transition. It remains notable that Mud Creek failed catastrophically during the extreme wet season of 2017 after > 8 years of slow-motion while numerous slow-moving landslides in Big Sur and elsewhere in the California Coast Ranges accelerated, but continued sliding slowly^31,42,59,80^. More recently, there have been several smaller and shallower landslides that have impacted CA 1 during the wet 2023 and 2024 years (https://www.usgs.gov/programs/cmhrp/news/usgs-remote-sensing-data-tracks-big-sur-landslides-2024), but none that match Mud Creek in scale.

Slow-moving landslides are thought to be controlled by rate-strengthening behavior that can be either due to intrinsic material properties^87,88^ or mechanical-hydrological feedbacks due to dilation^75,89–91^. Recent field observations by Finnegan & Saffer^87^ determined that the Minor Creek and Oak Ridge slow-moving landslides occurring in the Franciscan mélange, the same rock unit as Mud Creek, are predominantly composed of rate-strengthening material. However, as the rock unit name implies, the “mélange” is a mix of rock types, some of which may exhibit rate-weakening properties. In fact, the Oak Ridge landslide has also exhibited stick slip events^92^ that may arise from motion on velocity-weakening patches. Our results seem to support the interpretation by Finnegan & Saffer^87^ that slow-moving landslides in Franciscan mélange are primarily controlled by rate-strengthening friction but may contain rate-weakening patches (i.e. intrinsic material properties) that could lead to stick slip, or possibly catastrophic failure depending on the size of the slip patches relative to the critical nucleation size^93^. Mechanical-hydrologic feedbacks from shear dilation and compaction may also contribute to the changing stability regime^89^. Specifically, the capacity for a shear zone to dilate and maintain the negative transient perturbations to pore-water pressure that enhance shear strength likely diminishes with accumulated slip^90^. While more work is needed, our work provides new insights into the mechanisms that caused catastrophic failure. The strain rate maps (Fig. 3) showed that shear localized to a different sliding surface during the accelerating phase approximately ~ 1 month prior to failure and the failure forecast analysis (Figs. 4 and 5) showed a hyperbolic trend, which is suggestive of crack growth (rather than crack nucleation). Thus we hypothesize that crack growth and subsequent shear localization could be the weakening mechanisms that ultimately led to the runaway failure^94,95^.

Looking towards the future, we are optimistic that landslide failure forecasting based on displacement monitoring will continue to improve in ways that will enhance early warning systems and risk assessment protocols. Since the 2017 Mud Creek landslide, there have been improvements and advances in satellite systems, remote sensing tools and methods, and data access and analysis. For example, both image georeferencing accuracy and the frequency of PlanetScope acquisitions have increased since 2017 that will further reduce uncertainty and improve landslide displacement measurements^18,96^. There are also an increasing number of new freely available satellite SAR systems that will provide free and open access data such as the soon-to-be launched Sentinel-1D satellite, and recently launched NASA-ISRO SAR (NISAR) and Sentinel-1C satellite. Alongside these data are the development of large scale operational projects that provide the measurements of surface displacement time series needed to generate data over all landslide areas. Projects like the Observational Products for End-Users from Remote Sensing Analysis (OPERA), managed by the NASA Jet Propulsion Laboratory and sponsored by the U.S. Satellite Needs Working Group, are generating operational high resolution InSAR displacement time series from Sentinel-1 and NISAR data over North America (https://www.jpl.nasa.gov/go/opera/). These data can be directly downloaded and analyzed in near real time for landslide analysis and forecasting, removing the processing burden from individual users. Similar large scale processing systems are needed for pixel tracking of SAR and optical images. These systems are under development and are currently available in an on-demand mode, such as the Multiple-Pairwise Image Correlation toolbox for processing OPTical images (MPIC-OPT) service^36^ https://en.poleterresolide.fr/on-demand-processing/#/optic). While PlanetScope data is not yet viable for operational detection of new landslides at the regional-scale due to data quotas and commercial license constraints, it remains highly valuable for landslide monitoring on a case-by-case basis. Looking ahead, as data availability increases, there is a growing need for enhanced tools to support landslide detection and forecasting. Recent advances in machine learning and network science^76,84,85,97^ offer two promising approaches that are designed to handle big data that may further facilitate identification of landslide failure.

## Conclusions

In this study, we reexamined the precursory motion of the 2017 Mud Creek landslide in Big Sur, CA, USA using new and previously published data. Our results demonstrated that pixel tracking time series from PlanetScope optical images successfully captured the runaway acceleration leading to catastrophic failure—motion that had not been observed with satellite InSAR data. Using a statistical approach for the classic failure forecast method, we found that the acceleration toward failure was well described by a hyperbolic model. Additionally, 2D strain rate analysis revealed the formation of a new shear surface during the final month before failure, which may have been the key weakening mechanism that ultimately triggered collapse. We also used 3D surface velocity measurements derived from multi-temporal lidar and InSAR to invert for the subsurface geometry of Mud Creek prior to failure. Comparing our inversion results with pre- and post-catastrophic failure lidar DEMs, we found that our approach captured the magnitude and spatial pattern of landslide thickness to first-order, and supported our interpretation of new shear surface formation. Our findings show that multi-sensor remote sensing techniques should be used in concert to correctly identify, monitor, and forecast landslides. To improve failure forecasting from remote sensing at regional scales, operational systems and big data analysis tools are needed to identify landslides, blend datasets acquired from different sources, track landslide motion, and identify any key changes in landslide behavior. Continued research of similar landslides worldwide will enhance our understanding of the mechanisms driving slope instability, in particular those that enable the transition from slow-to-fast motion. Multi-method and multi-sensor satellite, airborne, and ground-based data approaches are the best way forward to predict the timing, location, and extent of landslides worldwide.

## Methods

### Surface deformation

#### Satellite and airborne synthetic aperture radar interferometry

The satellite and airborne InSAR data used in this study were previously published in Ref.^59^. We did not modify this published dataset. A brief summary is provided below. For detailed description of the InSAR methodology refer to the Methods section of Ref.^59^.

InSAR data acquired by the Copernicus Sentinel-1 A/B satellites and the NASA/JPL Uninhabited Aerial Vehicle Synthetic Aperture Radar (UAVSAR) aircraft were processed to interferograms using the InSAR Scientific Computing Environment (ISCE) software package^98^. The Sentinel-1 data were collected from 2015 to 2017 and the UAVSAR data were collected from 2009–2017. The full list of interferograms used in the analysis are listed in Supplementary Tables 1, 2, and 5 of Ref.^59^. All interferograms were referenced to a nearby stable (i.e., not-moving) reference point (Fig. 1). Reference^59^ used ascending and descending geometries from Sentinel-1, and an east–west flying UAVSAR line to invert for a true 3D surface velocity field (Fig. 1).

The Small Baseline Subset (SBAS) method^99,100^ with the Generic InSAR Analysis Toolbox (GIAnT) software^101^ was applied to the Sentinel-1 satellite data to measure the time-dependent motion of the landslide. We re-examined the time series on the descending track 42 that had the line-of-sight projected onto the downslope direction. The result was a cumulative displacement time series that is used to examine the landslide behavior leading up to collapse. The mean uncertainty for the Sentinel-1 time series was reported as 0.006602 ± 0.006586 mm/yr (± 1 standard deviation).

#### Pixel offset tracking

The pre-collapse motion of the Mud Creek landslide was investigated using pixel offset tracking. Pixel offset tracking methods provide 2D measurements (i.e., east–west, north–south) and are better suited for measuring large displacements that are ~ 1/10 of a pixel size. We measured pixel offsets from 3 m pixel PlanetScope optical satellites and 1 m pixel airborne lidar data, both of which can be used to measure displacement at the 10s of centimeter-scale (see *Data and software availability*)**.**

#### Pixel offset tracking with PlanetScope optical imagery

The PlanetScope constellation provided 42 images at the Mud Creek site between August 30, 2016 and May 20, 2017. This time period was selected because it spanned a period of known slow motion leading up to runaway failure. Unfortunately, however, many images during the rainy season of 2017 were obscured by cloud cover. We selected 22 cloud-free images covering the Mud Creek landslide (see *Data and software availability*). We quantified the 2D displacement time series using the green band of the PlanetScope images and the approach developed in Ref.^102^ and more recently applied to similar data in Ref.^18^. The approach uses the following steps: (1) correlate all image pairs using Mic-Mac software ^103^ that follows a hierarchical matching scheme using normalized cross-correlation over windows of 7 pixels minimum size, (b) mask out low correlation coefficient (CC) values (CC < 0.7), (c) mosaic correction by removing the median value of the along-track stacked profile taking into account only stable areas, (d) least square inversion weighted by the inverse of the time-difference between pairs as realized in Ref.^104^, giving more weight to the short time baselines, (e) correction of illumination effects. The uncertainties of the displacement fields per date were then estimated by the Normalized Median Absolute Deviation (NMAD) indicator over the stable areas. The uncertainty is between 28 and 103 cm, with a mean of 58 cm.

#### Pixel offset tracking and difference of DEM with airborne lidar

We also quantified the pre-catastrophic failure motion of Mud Creek using pixel offset tracking measured from 1 m USGS 3DEP lidar datasets (see *Data and software availability*). Airborne lidar data were acquired by the USGS 3DEP program along the Big Sur Coast in August 2010, May 2016, and March 2018. To prepare the lidar data sets for pixel tracking, we first aligned the last returns (“bare earth”) point clouds using the iterative closest point algorithm^105^, applied to points on presumably stable terrain surrounding the landslide. Two different subsets of the 2010 point cloud were poorly georeferenced, so we aligned them individually to the 2016 data before merging them into a single 2010 point cloud. The final RMSE of the alignment was 0.22 m, comparable to the uncertainty inherent in lidar data, and residuals were normally distributed around zero, indicating a satisfactory alignment. The point clouds were then interpolated to 1 m DEMs using the natural neighbor method^106^.

Because lidar measures ground surface elevation, we are able to construct 3D displacement fields from the lidar DEMs. We measured horizontal displacements using the phase correlation method described by Ref.^53^. We applied a 16 m × 16 m window centered on each pixel in the first DEM and computed offsets within a 32 m × 32 m search window centered on that same pixel in the second DEM. These window sizes were chosen to be large enough to produce a nearly continuous displacement field, without losing information near the edges of the lidar datasets. The minimum detectable velocity, defined here as 2σ (95% confidence interval) of the distribution of apparent velocities on stable terrain surrounding the landslide was 0.1 m/yr. Physically inaccurate motion identified as those pixel offsets exceeding several meters and appearing randomly oriented, including uphill motion, on both the landslide and stable parts of the study area was masked. Lastly, pixels with masked velocities within the landslide boundary were interpolated from surrounding velocities using the natural neighbor method to produce a complete estimation of the horizontal velocity field. Vertical velocities were calculated for each pixel by DEM differencing (Eulerian reference frame).

We also measured topographic change after the catastrophic failure of Mud Creek using a DEM of Difference (DoD) approach. We differenced the 2016 lidar DEM with the 2018 lidar DEM. We then identified ground returns from the point clouds using the Simple Morphological Filter (SMRF) described by Ref.^107^. The resultant ground points were then converted to a raster by taking the minimum values of points within a 1 m resolution grid. We used five control areas to assess the error in the processed DEMs between the two datasets. Two of these control areas, of around 500 m^2^ each, were on the road to the North and South of the landslide. The others were between 75 and 200 m^2^ distributed on a dirt road that ascends the hillslope to the south of the site. The differences in elevations on these surfaces were approximately normally distributed, with the post event DEM being slightly higher than the pre-event DEM. The median difference was 8 cm, with the 5th, 25th, 75th and 95th percentile differences at 1, 6, 11, and 16 cm, respectively. We note that the 2018 lidar survey was collected about 1 year after the catastrophic failure of Mud Creek, during which time road construction had begun and there had also been significant reworking of the Mud Creek failure surface. We used a vertical differencing (e.g., the M23C algorithm; Ref.^108^ rather than a differencing normal to the pre-failure surface to maintain consistency with the landslide thickness inversion.

#### Strain rate calculations

We perform strain rate calculations using the 2D pixel offset tracking time series of PlanetScope and apply the method of Ref.^109^. The 2D deformation rate tensor is defined as

$$\dot{D}=\left[\begin{array}{cc}{\dot{D}}_{xx}& {\dot{D}}_{xy}\\ {\dot{D}}_{yx}& {\dot{D}}_{yy}\end{array}\right]$$

$$=\left[\begin{array}{cc}{\dot{D}}_{xx}& \frac{1}{2}\left({\dot{D}}_{xy}+{\dot{D}}_{yx}\right)\\ \frac{1}{2}({\dot{D}}_{xy}+{\dot{D}}_{yx})& {\dot{D}}_{yy}\end{array}\right]+\left[\begin{array}{cc}0& \frac{1}{2}\left({\dot{D}}_{xy}-{\dot{D}}_{yx}\right)\\ -\frac{1}{2}({\dot{D}}_{xy}-{\dot{D}}_{yx})& 0\end{array}\right]$$

1 $$=\left[\begin{array}{cc}\frac{{\partial V}_{E}}{\partial x}& \frac{1}{2}\left(\frac{{\partial V}_{E}}{\partial y}+\frac{{\partial V}_{N}}{\partial x}\right)\\ \frac{1}{2}\left(\frac{{\partial V}_{E}}{\partial y}+\frac{{\partial V}_{N}}{\partial x}\right)& \frac{{\partial V}_{N}}{\partial y}\end{array}\right]+\left[\begin{array}{cc}0& \frac{1}{2}\left(\frac{{\partial V}_{E}}{\partial y}-\frac{{\partial V}_{N}}{\partial x}\right)\\ -\frac{1}{2}(\frac{{\partial V}_{E}}{\partial y}-\frac{{\partial V}_{N}}{\partial x})& 0\end{array}\right],$$

where the deformation rate tensor, $\dot{D}$, is the sum of the strain rate (irrotational) matrix and rotational rate matrix, and ${\dot{D}}_{xx}=\frac{{\partial V}_{E}}{\partial x}$, ${\dot{D}}_{yy}=\frac{{\partial V}_{N}}{\partial y}$. We only consider deformation in 2D (horizontal strain and rotation) because all of the velocity measurements are on the surface. After computing the strain tensor, the principal strains are the eigenvalues of the matrix, and the orientation of the principal strains are the eigenvectors. Since strain is the derivative of velocity with distance, it amplifies measurement noise and can make the final product too noisy to interpret. To reduce measurement noise, we first smooth the pixel offset tracking results by a Gaussian moving window. We select the window size of 5 pixels with 5 pixels as the standard deviation. The dilation rate is the sum of the eigenvalues, which represents the change of area. Based on our definition, positive and negative values represent contraction rate and extension rate, respectively. The maximum strain rate is the difference of the eigenvalues. This value indicates the possible maximum shear strain rate in the strain rate system.

### Progressive failure and failure forecast method

Progressive material weakening and failure (in response to elevated stress) can be described by a fundamental law which relates the acceleration in a geophysical precursor $\Omega$ (such as displacement, strain, or number of earthquakes) to its rate by:

2 $$\frac{{d}^{2}\Omega }{d{t}^{2}}=K{\left(\frac{d\Omega }{dt}\right)}^{\alpha },$$

where $\alpha$ and $K$ are constants^110^. Equation (2) is commonly known as the Failure Forecast Method (FFM). For different values of $\alpha$, the expected evolution of the precursor rate with time takes different forms. In the general case that 1 < α < 2 solutions to Eq. (2) take the form of an inverse power-law increase in the mean rate of precursory signals with time^24^:

3 $$\frac{d\Omega }{dt}={k}_{pl}{\left({t}_{f}-t\right)}^{-p},$$

where the power-law exponent, $p=\frac{1}{\left(\alpha -1\right)}$ describes the non-linearity of the acceleration, and ${k}_{pl}$ reflects the absolute amplitude^83^. For the specific cases of $\alpha =1$ and $\alpha =2$, respectively, the acceleration takes the form of either an exponential:

4 $$\frac{d\Omega }{dt}={k}_{e}{e}^{\lambda \left(t-{t}_{0}\right)},$$

or hyperbolic:

5 $$\frac{d\Omega }{dt}={k}_{hyp}{\left({t}_{f}-t\right)}^{-1},$$

increase in rate with time, with corresponding amplitude terms ${k}_{e}$ and ${k}_{hyp}$, and where ${t}_{0}$ corresponds to the time of the start of the failure process. Equations (3) & (5) involve a singularity at a finite time, *t*_*f*_, corresponding to an infinite precursor rate, realization of a system-wide fracture and the percolation threshold, and often equated to the initiation of the eruption, earthquake, or landslide process^20^. Here we use the approach of Ref.^16^ and the displacement measured from pixel offset tracking of PlanetScope optical data to explore the failure forecast potential of the Mud Creek landslide.

Theoretical considerations suggest that $\alpha \approx 2$ is typical of rock failure and landslides^24,78^, in which case the inverse precursor rate is expected to decrease linearly with time to failure. This approach has been used to visualize and model the evolution in precursor (commonly ground deformation) rate, and proposed as a means to forecast the timing of future failure events^17,19,24,78^. However, the common practice of using least-squares regression to fit a straight line to inverse precursor rates fails to account for the complex error structure of such data, resulting in biased and inaccurate forecasts and parameter estimates^111^. Instead, Generalized Linear Models^111^, maximum-likelihood^112^ and Bayesian^113^ methods have been proposed as improved methods to estimate model parameters for volcanic and laboratory data. We used a Bayesian Markov Chain Monte Carlo (MCMC) modeling approach to analyze the accumulation of precursory deformation before the Mud Creek landslide.

We model the cumulative displacement as a function of time using a non-linear Gaussian regression. Each incremental data acquisition measures the displacement of the land surface, and we begin by assuming the deviations between the measured displacements and any systematic underlying temporal trend in displacements, are described by a Gaussian distribution with a fixed variance and are uncorrelated in time. This assumption is unlikely to be correct in detail, but the modeling results may still be useful.

We use a Bayesian approach to estimate model parameters and MCMC to estimate parameter posterior distributions from the point process likelihood function, implemented through PyMC^114^. Using a No-U-Turn Sampler (NUTS) and automatic differentiation variational inference (ADVI) initiation (see Dataset and Software Availability), good convergence is returned in 10,000 sample steps, and a 1000 sample ‘burn-in’ period. Bounded uniform prior distributions are used for all model parameters.

### Landslide thickness inversion

We estimated the thickness and volume of the slow-moving Mud Creek landslide using 3D surface velocity measurements and volume conservation. Our methodology follows the framework initially outlined by Ref.^52^ and later refined by Refs.^53^^,^^55^. This approach operates under several key assumptions: (1) the surface velocity serves as a reasonable proxy for the depth-averaged velocity during the study period; (2) the landslide moves along a single sliding surface of constant geometry over the time interval of velocity measurements; (3) direct erosion or deposition on the landslide surface is minimal; and (4) the density of the landslide material remains uniform and constant. While landslides can sometimes deviate from these conditions, they are appropriate for this study because similar landslides in California exhibit surface velocities that closely approximate depth-averaged velocities, with evidence of a narrow shear zone^68,115^; the landslide was continuously active within fixed spatial boundaries during the period of 3D displacement measurements, suggesting motion along a consistent slip surface; any direct erosion or deposition was likely limited to small gully systems covering only about 1% of the landslide’s surface, meaning their effect on volume estimates was negligible; and changes in thickness caused by variations in material density due to dilation, compaction, or other processes were likely on the order of centimeters or less^53,54,89,115^, which is small relative to surface velocity gradients and therefore had minimal influence on the 3D velocity measurements. Consequently, for a landslide with stable density and no significant surface modification, the principle of volume conservation is reasonable.

To perform thickness inversions, we used our completely independent 3D surface velocity measurements from InSAR and lidar (Fig. 1a,b). For the lidar-based inversion (which quantifies surface displacements in an Eulerian reference frame)

6 $$\frac{\partial z}{\partial t}=-\nabla \bullet \left(f{u}_{surf}h\right),$$

where $\frac{\partial z}{\partial t}$ is the vertical velocity from lidar differencing, *f* is a proportionality constant that relates the depth-averaged velocity to the surface velocity, *u*_surf_ is the horizontal surface velocity from lidar pixel tracking, and *h* is the landslide thickness. The 3D InSAR data are in a Lagrangian reference frame^53,54^, so Eq. (6) is redefined as

7 $${v}_{ud}=-\nabla \bullet \left(f{u}_{surf}h\right)+{u}_{surf}\bullet \nabla {z}_{surf},$$

where ${v}_{ud}$ is the vertical component of the 3D displacement vector measured with InSAR, *z*_surf_ is the surface elevation measured from the DEM. Since there were no subsurface observations to constrain the depth-averaged velocity, for simplicity we assume that *f* = 1, i.e., Mud Creek moves as a block. Previous work on slow-moving landslides in California and elsewhere^116–118^ have shown that landslides in similar materials move as essentially a rigid plug above a narrow shear zone. Assuming instead that *f* < 1 increases the magnitude of the landslide thickness proportionally everywhere, but does not affect the spatial pattern of variations in depth ^52^.

Equations (6) and (7) are discretized using centered finite differences and rearranged as a system of linear equations where the thickness *h* can be found by minimizing the value of

8 $${\left|Xh-b\right|}^{2}+{\alpha }^{2}{\left|{\nabla }^{2}h\right|}^{2},$$

where *X* is a diagonally dominant matrix containing the horizontal velocity data, *b* is defined as ${u}_{surf}\bullet \nabla {z}_{surf}-{v}_{ud}$ for the Lagrangian reference frame, or as $\frac{\partial z}{\partial t}$ for the Eulerian reference frame, and *α* serves as a damping parameter to regularize the ill-posed inverse problem. Because both *X* and *b* contain data with inherent uncertainties, and the choice of *α* introduces bias, accurately quantifying the total uncertainty in the estimated thickness model is challenging. Reference^55^ provided minimum uncertainty estimates using the same methods used in this work and reported a mean value of ± 2.4 m. Uncertainty can also vary spatially and was generally lowest throughout the center of the landslide (faster moving area), and increased near its margins. To determine an optimal regularization level, we explore a broad range of α values from 10^–3^ to 10^1^, selecting the best option using the Generalized Cross-Validation method. The datasets are resampled to a uniform 10 × 10 m grid, and the thickness inversion is carried out using MATLAB with the CVX optimization package^119^. More details and derivations equations for the inversions for the lidar and InSAR-based approaches are described in Ref.^53^    and Ref. 55, respectively.

Finally, it is important to clarify that the thickness estimates apply to the actively deforming regions of the landslide. This means that surface deformation must be detectable for thickness to be determined. Any landslides or portions of them that do not exhibit detectable movement are assigned a thickness of zero. As a result, the term “landslide thickness” specifically refers to the “active thickness” inferred during the study period.

## Supplementary Information

Supplementary Information 1.

Supplementary Information 2.

Supplementary Information 3.

## Data Availability

Copernicus Sentinel-1 level-1 images processed by the ESA are available through the Alaska Satellite Facility (ASF) Distributed Active Archive Centers (DAAC) for free (https://search.asf.alaska.edu/). The full list of Sentinel-1 interferograms from Handwerger et al.^59^. Lidar digital elevation models are available via the USGS 3D Elevation Program and are provided by OpenTopography (data set name: “ARRA-CA CentralCoast-Z4 2010”, “USGS LPC CA WestCoastElNinoUTM10 2016 LAS 2017”, “CA FEMA Z4 B2 2018”) and may be downloaded at http://www.opentopography.org. NASA/JPL UAVSAR Single-Look Complex (SLC) data are freely available at https://uavsar.jpl.nasa.gov/. Precipitation data is provided by Parameter-elevation Regressions on Independent Slopes Model (PRISM) available at https://prism.oregonstate.edu/. Precipitation time series for Mud Creek landslide is taken at the centroid location. All the PlanetScope images are publicly available on the PlanetScope webserver (https://www.planet.com/explorer/). PlanetScope images were provided by Planet’s Education and Research Program to P.L. PlanetScope imagery time series can be viewed here https://www.planet.com/stories/mud_creek_aug2016-may2017_v2-YRQCAjbIg.
