# Supplementary material for: Multi-sensor remote sensing captures geometry and slow-to-fast sliding transition of the 2017 Mud Creek landslide
Source: Sci Rep. 2025 Aug 14;15:29831. doi: 10.1038/s41598-025-11399-8 (PMC12354922; doi:10.1038/s41598-025-11399-8)
Supplement: Supplementary file 1 — Supplementary Information 1. [file 41598_2025_11399_MOESM1_ESM.docx]

Supplementary Information for

Multi-Sensor Remote Sensing Captures Geometry and Slow-to-Fast Sliding Transition of the 2017 Mud Creek landslide

Alexander L. Handwerger^1,2,*^, Pascal Lacroix^3^, Andrew F. Bell^4^, Adam M. Booth^5^, Mong-Han Huang^6^, Simon M. Mudd^4^, Roland Bürgmann^7^, and Eric J. Fielding^1^

1Jet Propulsion Laboratory, California Institute of Technology, Pasadena, 91109, USA,

2Joint Institute for Regional Earth System Science and Engineering, University of California, Los Angeles, 90095, USA.

3ISTERRE, University Grenoble Alpes, University Savoie Mont Blanc, CNRS, IRD, UGE, ISTerre, Grenoble, France.

4School of GeoSciences, University of Edinburgh, Edinburgh EH9 3FE, UK.

5Department of Geology, Portland State University, Portland, OR, 97207, USA

6Department of Geology, University of Maryland, College Park, MD, USA.

7Department of Earth and Planetary Science, University of California, Berkeley, CA, 94720, USA

**
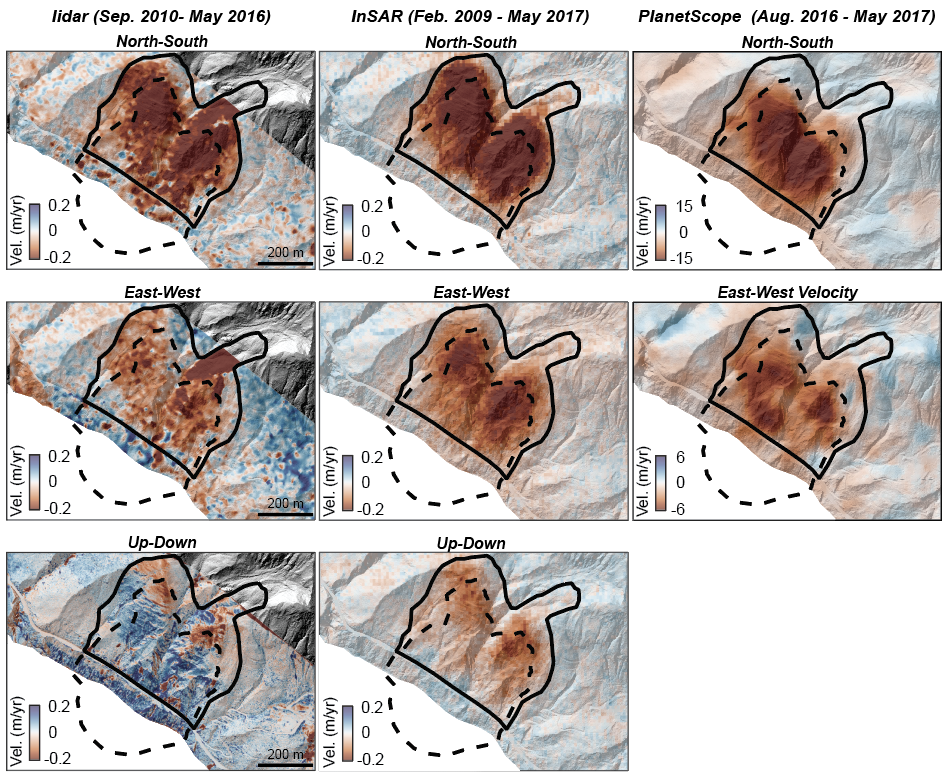
**

**Supplementary Figure 1. Mapped landslides and geologic map**. Surface velocity of Mud Creek landslide. North-South, East-West, and Up-Down velocity components are displayed. Velocity is measured from (left column) lidar pixel tracking and difference of DEM (Sep. 2010 - May 2016), (middle column) InSAR (Feb. 2009 - May 2017), (right column) PlanetScope pixel tracking (Aug. 2016 - May 2017). Velocity maps and velocity vectors are draped over the pre-catastrophic failure hillshade of topography. Solid black line shows the pre-catastrophic failure landslide boundary. Dashed black line shows the catastrophic failure area including the landslide scar and deposit. Note the large difference in velocity magnitude between lidar/InSAR and PlanetScope, due to the latter capturing the rapid acceleration prior to failure (see Fig. 2). InSAR data were previously published in Handwerger, Huang, et al. (2019).

**
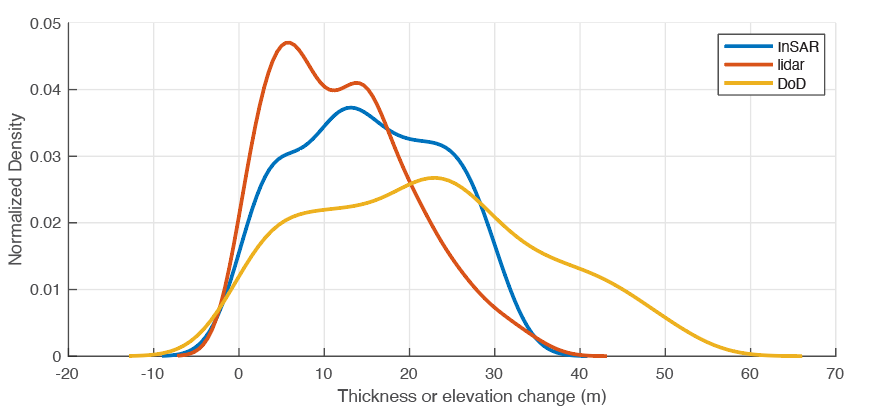
**

**Supplementary Figure 2. Kernel density plot of inverted thickness and elevation change**. Inverted thickness estimates from lidar and InSAR and elevation change (positive here means elevation loss) measured from difference of DEM.

**Supplementary Table 1. Landslide kinematic metrics.** Average velocity and time series displacement metrics derived from lidar, InSAR, and PlanetScope.

**Supplementary Table 2. Landslide geometry metrics.** Area, length, width, thickness, and volume.
